# Associations of sport participation, muscle-strengthening exercise and active commuting with self-reported physical fitness in school-aged children

**DOI:** 10.3389/fpubh.2022.873141

**Published:** 2022-07-22

**Authors:** Chongyan Shi, Sitong Chen, Lei Wang, Jin Yan, Kaixin Liang, Jintao Hong, Hejun Shen

**Affiliations:** ^1^School of Physical Education and Humanity, Nanjing Sport Institute, Nanjing, China; ^2^Institute for Health and Sport, Victoria University, Melbourne, VIC, Australia; ^3^School of Physical Education and Sport Training, Shanghai University of Sport, Shanghai, China; ^4^Centre for Active Living and Learning, University of Newcastle, Newcastle, NSW, Australia; ^5^College of Human and Social Futures, University of Newcastle, Newcastle, NSW, Australia; ^6^School of Psychology, Shenzhen University, Shenzhen, China; ^7^Shanghai Research Institute of Sports Science (Shanghai Anti-Doping Agency), Shanghai, China

**Keywords:** sports participation, muscle strengthening exercise, active school travel, self-reported physical fitness, China

## Abstract

**Background:**

Numerous studies suggest a positive association between physical activity and physical fitness in schoolchildren. However, little is known about some neglected forms of physical activity and their associations with physical fitness. This study was conducted *via* a self-reported questionnaire, owing to the COVID-19 pandemic in many regions in China.

**Purpose:**

This study explores the associations between participating in sports, muscle-strengthening exercises, and active commuting with self-reported physical fitness assessed by the International Fitness Scale (IFIS).

**Methods:**

A total of 3,807 study participants (ages 11–17) from 12 public schools in South-eastern China were recruited, with 2,407 providing valid data on variables for analysis. Study participants were asked to self-report their sociodemographic factors (i.e., sex, grade, age), participation in sports (never, 1–3 times per month, 1–2 times per week, and 3 or more times per week), muscle-strengthening exercise (0–7 days) and active commuting (0–5 days). Generalized linear models were used to explore the associations between sports participation, muscle-strengthening exercise, and active commuting with self-reported physical fitness (comprising general physical fitness, cardiorespiratory fitness, muscular strength, speed and agility, and flexibility). A total of 2,407 children and adolescents with a mean age of 13.82 (±2.1) years were included in the study's final analysis.

**Results:**

The study found no significant association between active commuting and physical fitness. Regarding participating in sports and muscle-strengthening exercises, positive, significant associations were found, which showed that a higher frequency of participating in sports and more participation in muscle-strengthening exercises are associated with improved physical fitness.

**Conclusion:**

This study offered evidence on the roles of some aspects of physical activity in physical fitness. To promote health in children and adolescents, they should be encouraged to participate in more sports and engage in muscle-strengthening exercises.

## Highlights

- Physical fitness levels should be improved considerably in Chinese children and adolescents.
- Sports participation and muscle-strengthening exercise (MSE) were associated with higher levels of self-reported physical fitness.
- Active commuting might not be a contributor to self-reported physical fitness in Chinese children and adolescents.

## Introduction

Physical fitness is defined as a set of attributes that people have or achieve while maintaining physical activity (1, 2). It is a well-recognised marker of an individual's health status (3–5). Physical fitness is classified as health-related and skill-related fitness (6). Five components compromise health-related physical fitness (HRPF): cardiorespiratory fitness, body composition, muscular strength, muscular endurance, and flexibility (6). Much evidence has demonstrated the importance and health benefits of higher levels of HRPF in children and adolescents (7, 8). For example, adolescents with higher levels of HRPF have a reduced risk of cardiovascular disease in later life (9). Cardiorespiratory fitness in adolescents is also directly associated with mental wellbeing and quality of life (10, 11). Also, higher cardiorespiratory fitness is positively associated with academic performance among adolescents (12). Muscular fitness (e.g., strength and endurance) is associated with lower risks of adiposity and cardiometabolic parameters and greater bone health in later life (13, 14). Because of the significance of HRPF, it is important to identify more effective ways to encourage it in children and adolescents. Nevertheless, synthesized data shows that lower levels of HRPF have been observed over the past two decades in children and adolescents (15).

Participating in regular and sufficient physical activity is associated with greater levels of HRPF in children and adolescents (7, 8). However, these studies focused on the overall levels of physical activity instead of different types of physical activity. According to earlier studies, adolescents can participate in many types of physical activity, including some that have been shown to be significantly associated with health outcomes and fitness components (8, 16, 17). For example, among the different types of physical activity, sports participation, muscle-strengthening exercise (MSE), and active commuting (AC) can all be organised frequently in school settings, while AC can happen daily. These two types are essential components of global surveillance of active lifestyles (18, 19). In terms of MSE, the World Health Organization recommends that young people (aged 11–17) should engage in it at least three times a week (20). Moreover, a large body of evidence indicates that participating in sports, MSE, and AC are associated with HRPF in adolescents (21–23).

Participating in sports is considered crucial to promoting positive health outcomes in children and adolescents (24, 25). Evidence has demonstrated higher levels of HRPF can be gained from participating in sports (26). A systematic review indicated that participating in organized sports was positively associated with muscular fitness among adolescents. AC plays a prominent role in preventing the risk of mental disorders (27, 28) and may reduce risks associated with poorer HRPF levels (21, 23). MSE is negatively associated with mental health disorders in adolescents and it can also promote muscular fitness (29). This evidence illustrates the importance of participating in sports, MSE, and AC for young people and promoting their health.

Studying the association between different types of physical activity and fitness helps design efficient and contextual fitness or health promotion plans. However, these associations remain rare in literature, limiting researchers' understanding of fitness promotion. It is, therefore, valuable to explore the associations of participating in sports, MSE, and AC with HRPF.

With the COVID-19 pandemic still active in many Chinese regions, assessing HRPF in adolescents using field-based assessment (such as a shuttle run for cardiorespiratory fitness) is unrealistic. Therefore, it is necessary to find an alternative to assess HRPF in adolescents. The International Fitness Scale (IFIS) is an option that can assess adolescents' HRPF simply and conveniently (30). The IFIS was developed in 2011 and much evidence has indicated that it is a reliable and valid instrument to assess HRPF in adolescents (30), with many studies confirming the psychometric properties of the IFIS in adolescents from various countries (31, 32). Unfortunately, evidence linking the benefits of participating in sports, MSE, and AC with self-reported HRPF is very rare, but this makes it worth studying.

To fill the research gap, this study, therefore, aims to explore the links between participating in sports, MSE, and AC (three different types of physical activity) with self-reported HRPF using a sample of Chinese adolescents.

## Methods

### Study design and participants

This study was a cross-sectional survey conducted between March and October in south-eastern China. 12 public schools in four cities in the South-eastern region were contacted, comprising 5 elementary schools, 5 middle schools and 2 high schools. In each school, 1–3 classes of each grade were randomly selected by a contact assigned to each school. This procedure recruited the initial sample comprising 3,807 children and adolescents (ages 11–17). Study participants providing information on variables of interest were included in this study, while those who did not report data on any variables (e.g., independents, outcomes and covariates) of interest that this study needed were excluded from the initial sample. For this study and further analysis, only 2,407 study participants were included as they provided valid data on variables this study needed. All the children and adolescents involved in the study, as well as their parents or guardians, were specifically advised that participation was completely voluntary. The study protocol and procedure were approved by the Institutional Review Board (IRB) of the Shanghai University of Sport with a Grant Number of 102772021RT071.

### Measures

#### Independent variables (sports participation, MSE, AC)

Sports participation was measured by one question about the participation in organized sports and/or programs over the past 12 months. Participants were required to answer the frequency of sports participation in one week, with answer options of *(1) Never, (2) 1–3 times per month, (3) 1–2 times per week, and (4) 3 or more times per week*. This item has demonstrated good reliability and validity in assessing sports participation of children and adolescents (33).

MSE was assessed by the following question: “*In the past week, how many days did you engage in exercise to strengthen or tone the muscle, such as push-ups, sit-ups, or lifting weights?*” The possible responses were: *0* = *none, 1* = *1 day, 2* = *2 days, 3* = *3 days, 4* = *4 days, 5* = *5 days, 6* = *6 days, and 7* = *7 days*. This measure has been confirmed as reliable and valid in assessing MSE among Chinese children and adolescents (34). Based on the recommendation of the World Health Organization, participants who responded for 3 days or more were considered to meet the MSE guideline, otherwise, they were classified as not meeting the guideline (20).

AC was assessed by two independent questions: (1) *On the weekdays, how many days did you go to school by walking, riding cycles, or other active ways*? and (2) *On the weekdays, how many days did go home after school by walking, riding cycles or other active ways?*. The answer options for both the questions were 0–5 days.

#### Outcome variable (physical fitness)

The International Fitness Scale (IFIS) was used to evaluate the self-reported levels of HRPF, using a five-point Likert scale (very poor, poor, average, good, and very good). The IFIS contains five components, including general physical fitness, cardiorespiratory fitness, muscular strength, speed and agility, and flexibility. The scale has demonstrated acceptable reliability and validity in adolescents (32). In addition to the study sample for further analysis, 544 Chinese children and adolescents were also recruited, who took part in a reliability study. The unpublished results indicated that the IFIS had acceptable reliability (weighted kappa: 0.42–0.52; coefficient of internal consistency of 0.72) in Chinese children and adolescents (35). There has been convincing evidence to demonstrate the validity of the IFIS in children and adolescents (30). Hence, it was deemed that the IFIS can be used as a workable and valid instrument to assess HRPF in study participants of this study.

#### Controlling variables

Information on study participants' age, sex, siblings, living with parents or not, grade, residence, father and mother education level, and perceived family affluence (0–10 scale) were measured by a self-reported questionnaire. These sociodemographic factors were treated as covariates in further statistical analysis. Besides, study participants' moderate to vigorous physical activity and recreational screen time was assessed by the measures derived from the Health Behaviour in School-aged Children survey, with acceptable reliability and validity in Chinese children and adolescents (36). Sleep duration was measured by the Pittsburgh Sleep Scale. Moderate to vigorous physical activity, recreational screen time, and sleep duration was dichotomized as binary variable based on the Canadian 24-h Movement Guidelines (37, 38).

#### Statistical analysis

**Figure 1:**
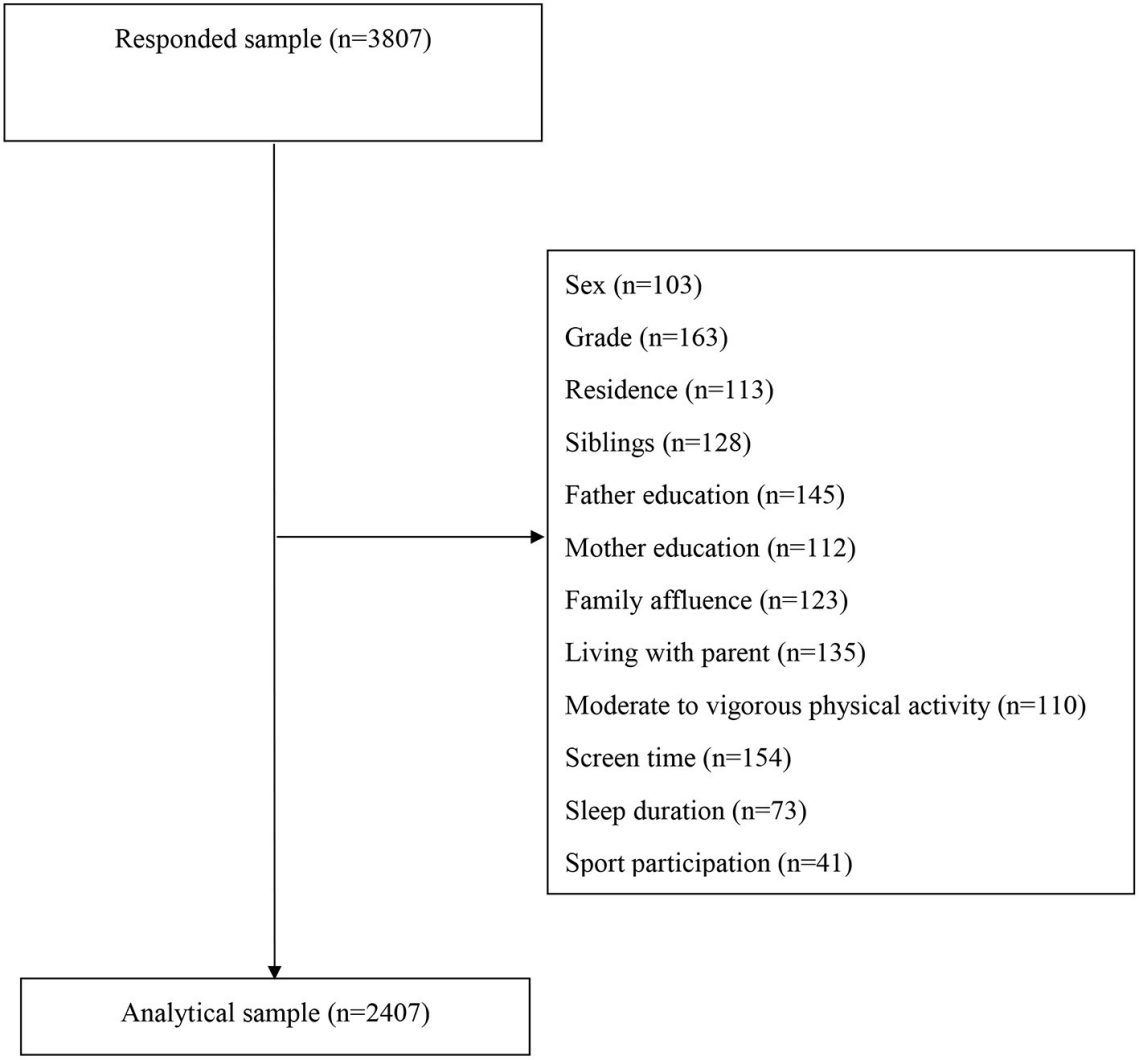
The procedure used for cleaning invalid and missing data in this study.

Prior to formal data analysis, missing data was coped with by using complete case analysis and the procedure can be found at Figure 1. All the statistical analysis was performed using SPSS 26.0 Version. Descriptive statistics (mean/standard deviation and percentage) were used to report sample characteristics. Mean with standard deviation was for continuous variables (e.g., age) while the percentage was for categorical variables (e.g., grade, residence). Partial correlation was used to explore the associations among sports participation, MSE, AC, and HRPF indicators after controlling for all the sociodemographic factors, moderate to vigorous physical activity, screen time, and sleep duration. To estimate the associations of sports participation (reference group: never), MSE (reference group: <3 times per week), and AC (0 days) with HRPF indicators, five separate models were established (model 1 for general physical fitness; model 2 for cardiorespiratory fitness; model 3 for muscular strength; model 4 for speed and agility; model 5 for flexibility). Entered in the model, sports participation, MSE, and AC were entered the model while controlling for all the other covariates. Generalized linear models with ordinal logistic regression were used to achieve the association estimation. The statistical significance was set up as *p* < 0.05.

## Results

Table 1 presents the sample characteristics. The mean age of all the study participants was 13.82 (±2.1), with boys accounting for 52.7%. More information on the study participants can be found in Table 1. In terms of sports participation, MSE, AC and self-reported HRPF indicators, 59.7% of the participants reported never engaging in any sports activities, whilst only 7.6% participated in sports more than three times per week. In addition, 23.4% met the MSE guideline. About 40% of study participants selected Active commuting for going to school or going home on weekdays.

**Table 1:** Sample characteristics of this study.

	**n/Mean**	**%/SD**
Age	13.82	2.1
**Sex**		
Boy	1,268	52.7
Girl	1,139	47.3
**Siblings**		
Yes	1,184	49.2
No	1,223	50.8
**Living with parents**		
Yes	2,018	83.8
No	389	16.2
**Grade**		
4	355	14.7
5	334	13.9
7	353	14.7
8	415	17.2
10	515	21.4
11	435	18.1
**Residence**		
Rural	277	11.5
Suburban	526	21.9
Urban	1,604	66.6
**Father education level**		
Middle school or below	623	25.9
High school	595	24.7
Undergraduate	772	32.1
Graduate	137	5.7
Unknown	280	11.6
**Mother education level**		
Middle school or below	770	32.0
High school	512	21.3
Undergraduate	732	30.4
Graduate	114	4.7
Unknown	279	11.6
Family affluence	5.09	1.5
**MVPA guideline**		
Not meet	2,250	93.5
Meet	157	6.5
**Screen guideline**		
Not meet	1,368	56.8
Meet	1,039	43.2
**Sleep guideline**		
Not meet	1,674	69.5
Meet	733	30.5
**Sport participation**		
Never	1,436	59.7
1-3 times per week	370	15.4
1-2 times per week	419	17.4
3 or more times per week	182	7.6
**MSE guideline**		
Not meet	1,844	76.6
Meet	563	23.4
**Active commuting on a weekday (go to school)**		
0	1,077	44.7
1 day	128	5.3
2 days	132	5.5
3 days	91	3.8
4 days	80	3.3
5 days	899	37.3
**Active commuting on a weekday (after school)**		
0	970	40.3
1 day	144	6.0
2 days	129	5.4
3 days	100	4.2
4 days	67	2.8
5 days	997	41.4
**Overall physical fitness**		
Very poor	78	3.2
Poor	297	12.3
Average	1,254	52.1
Good	581	24.1
Very good	197	8.2
**Cardiorespiratory fitness**		
Very poor	90	3.7
Poor	341	14.2
Average	1,140	47.4
Good	621	25.8
Very good	215	8.9
**Muscular strength**		
Very poor	85	3.5
Poor	380	15.8
Average	1,239	51.5
Good	549	22.8
Very good	154	6.4
**Speed/Agility**		
Very poor	63	2.6
Poor	305	12.7
Average	1,120	46.5
Good	667	27.7
Very good	252	10.5
**Flexibility**		
Very poor	166	6.9
Poor	530	22.0
Average	1,031	42.8
Good	491	20.4
Very good	189	7.9

*SD, standard deviation; MVPA, moderate to vigorous physical activity; MSE, muscle strengthening exercise*.

The results from the partial correlation between sports participation, adherence to the MSE guideline, Active commuting, and HRPF indicators are shown in Table 2 after controlling for all the covariates. Specific results indicate that sports participation was correlated with all the indicators of self-reported HRPF (*r* ranged from 0.16–0.24, *p* < 0.001). Adherence to the MSE guideline was also associated with all the indicators of self-reported HRPF (*r* ranged from 0.13–0.23, *p* < 0.001). Active commuting was correlated with HRPF indicators, except for flexibility. This result is also displayed in the association between active commuting for home and HRPF indicators.

**Table 2:** Partial correlation between sports participation, muscle strengthening exercise, active commuting and self-reported health-related physical fitness.

	**1**	**2**	**3**	**4**	**5**	**6**	**7**	**8**	**9**
1. Sports participation	1.00								
2. Muscle strengthening exercise	0.25^***^	1.00							
3. AC in weekday (school)	0.10^***^	0.06^**^	1.00						
4. AC in weekday (home)	0.08^***^	0.06^**^	0.80^***^	1.00					
5. General physical fitness	0.24^***^	0.21^***^	0.07^***^	0.06^**^	1.00				
6. Cardiovascular fitness	0.22^***^	0.20^***^	0.07^***^	0.07^**^	0.57^***^	1.00			
7. Muscular strength	0.21^***^	0.23^***^	0.04	0.05^*^	0.47^***^	0.43^***^	1.00		
8. Speed and agility	0.21^***^	0.19^***^	0.07^***^	0.05^*^	0.51^***^	0.43^***^	0.43^***^	1.00	
9. Flexibility	0.16^***^	0.13^***^	0.02	0.00	0.27^***^	0.24^***^	0.24^***^	0.27^***^	1.00

*AC, active commuting*.

* *p < 0.05*,

** *p < 0.01*,

*** *p < 0.001*.

**Figure 2:**
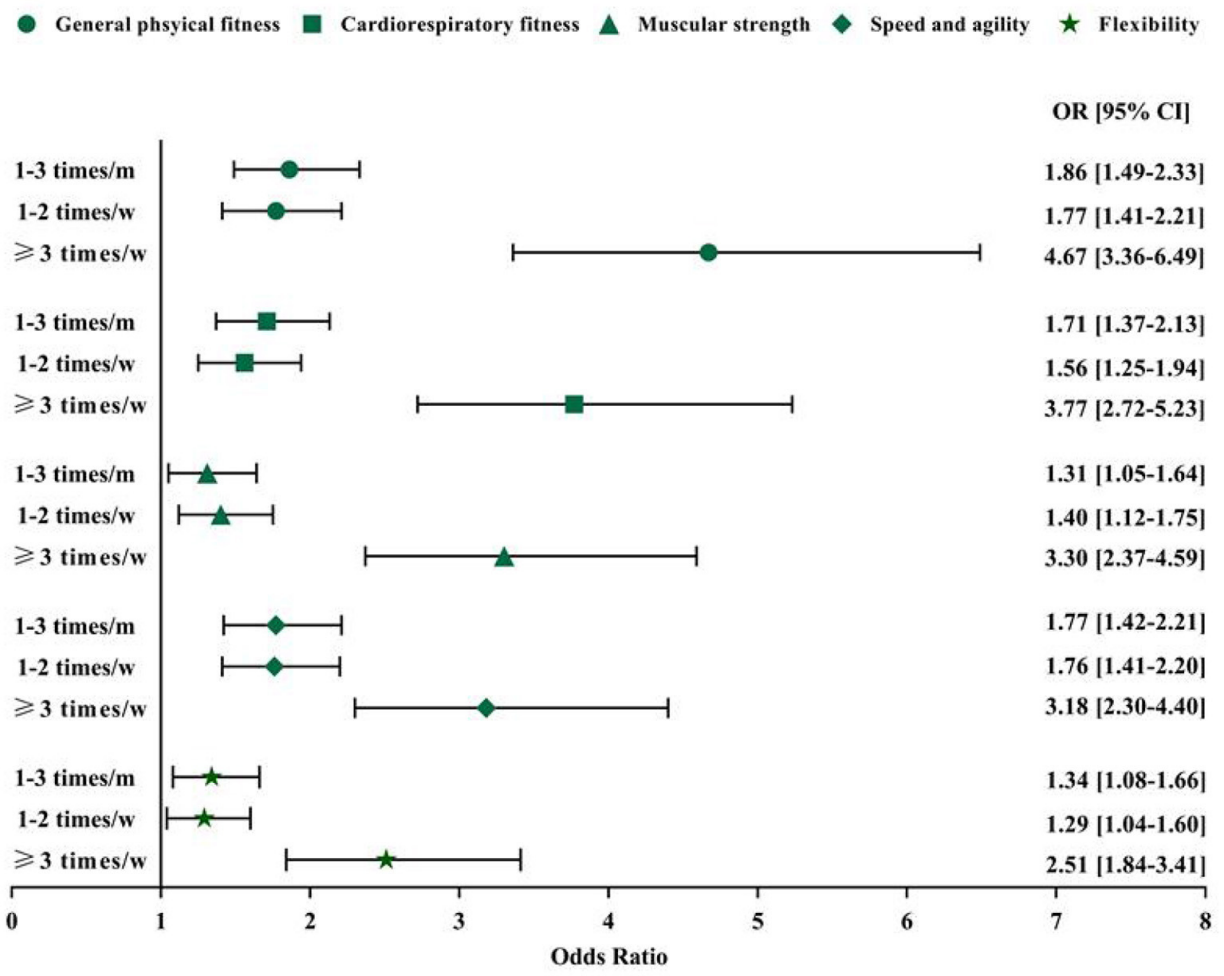
Association between frequency of sports participation and self-reported health-related physical fitness indicators. Model controlled for age, sex, siblings, living with parents or not, grade, residence, father and mother education level, and perceived family affluence, moderate to vigorous physical activity, recreational screen time and sleep duration. m, month; w, week; OR, odd ratio; CI, confidence interval. Reference group: never.

**Figure 3:**
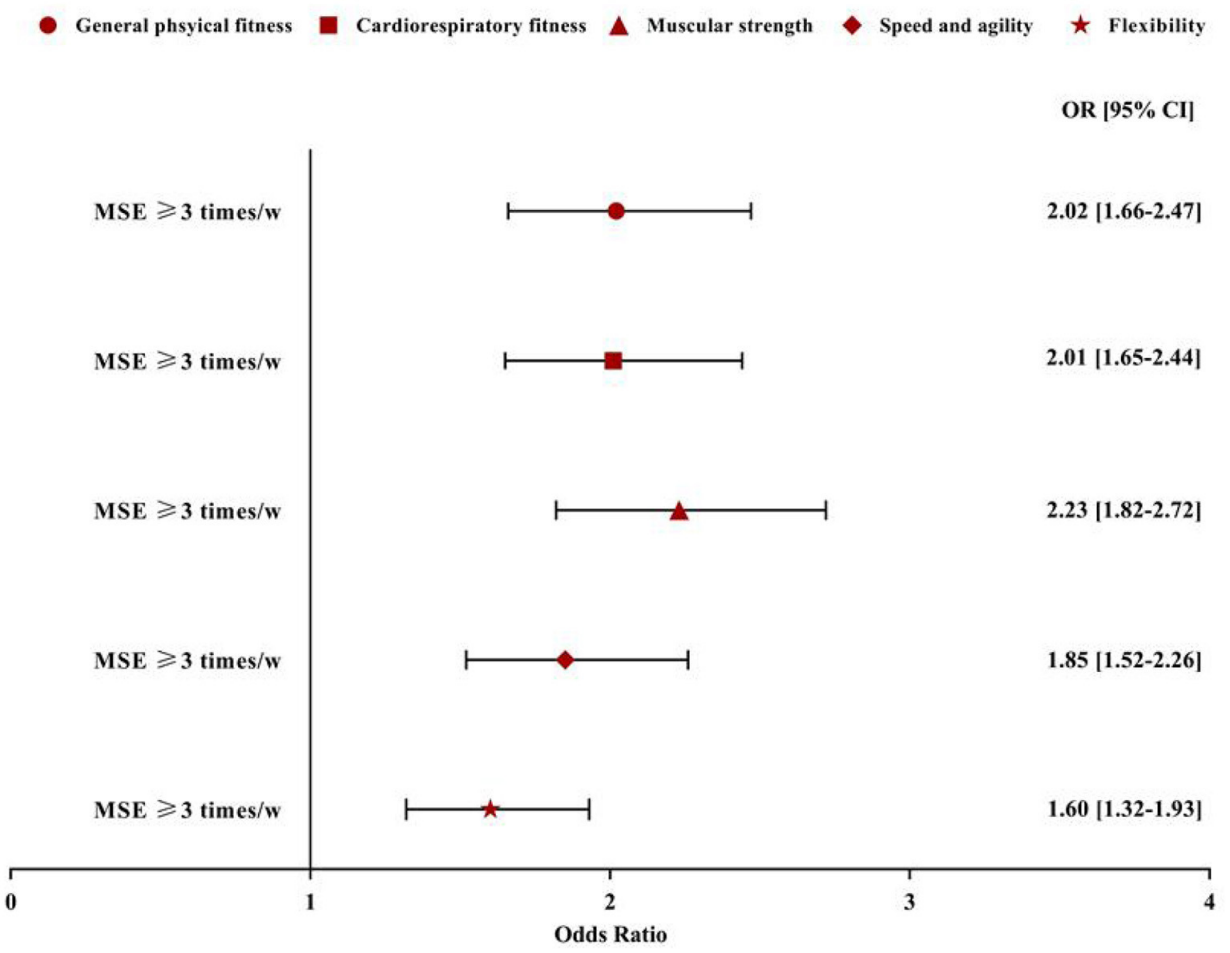
Association between muscle strengthening exercise and self-reported health-related physical fitness indicators. Model controlled for age, sex, siblings, living with parents or not, grade, residence, father and mother education level, and perceived family affluence, moderate to vigorous physical activity, recreational screen time and sleep duration. MSE, muscle-strengthening exercise; w, week; OR, odd ratio; CI, confidence interval. Reference group: no more than 3 times/week.

Following control of all the covariates in the current study results from the regression models suggested that sports participation (Figure 2) and adherence to the MSE guideline (Figure 3) were significantly associated with HRPF indicators in the study participants. Specifically, compared with participants who were never involved in sports participation, those reporting 1–3 times per month, 1–2 times per week, and 3 or more times per week had a greater likelihood of general physical fitness (OR = 1.86, 95% CI: 1.49–2.33; OR = 1.77, 95% CI: 1.41–2.21; OR = 4.67, 95% CI: 3.36–6.49). Similar results were also found in the association between sports participation and other HRPF indicators. However, no dose-dependent association was found between sports participation and HRPF indicators, except for muscular strength. As for the association between MSE and HRPF indicators, study participants meeting the MSE guideline were more likely to report higher levels of HRPF (OR for general physical fitness = 2.02, 95% CI: 1.66–2.47; OR for cardiorespiratory fitness = 2.01, 95% CI: 1.65–2.44; OR for muscular strength = 2.23, 95% CI: 1.82–2.72; OR for speed and agility = 1.85, 95%CI: 1.52–2.26; OR for flexibility = 1.60, 95% CI: 1.32–1.93). In the regression model, no significant association was found between Active commuting and HRPF indicators (all *p* > 0.05; data not shown) in the results.

## Discussion

The primary aim of the current study is to examine the association between several types of physical activity and self-reported physical fitness (e.g., overall physical fitness, cardiorespiratory fitness) in adolescents aged 11–17. It was found that participating in sports and MSE are positively associated with higher levels of self-reported physical fitness indicators in adolescents. However, AC was not found to be associated with any self-reported physical fitness indicators.

A growing body of evidence has confirmed the positive associations between participating in sports and HRPF indicators in children and adolescents. For example, the Physical Activity Health Longitudinal (PAHL) study reported that adolescents who regularly participate in sports had higher levels of physical fitness (field-based assessments) (26). A longitudinal study suggested that those who participate in sports outperformed those who do not when measuring cardiorespiratory fitness (field-based assessments) (39). Participating in sports was also favourably associated with muscular strength (self-reported questionnaire) (40).

This convincing evidence supports the present research findings. It is, therefore, expected that children and adolescents who participate in sport more frequently can increase their PA levels (41), which in turn improves levels of physical fitness indicators. However, compared with some other studies that investigated the context of participating in sports (e.g., in school or out-of-school) (42), the present study failed to categorise the contexts in which participation occurred. This limits the current study to further explore the contextual health promotion effectiveness of participating in sports in children and adolescents. Future studies should fill the research gaps. Regarding the associations between participating in sports and physical fitness indicators (except for muscular strength), a no dose-response association was found, which may go beyond expectations. A possible reason for this may be due to measurement bias resulting from self-reported measures on sports participation and physical fitness indicators. Of note, odds ratios for the association between participating in sports three or more times and physical fitness indicators were larger than other levels of sports participation. This may imply that participating in sports at a specific frequency can help improve physical fitness indicators in children and adolescents.

As an important component of physical activity, MSE has been confirmed to be associated with a variety of health benefits (43, 44), including promoting mental health (45, 46) and physical fitness improvement (47, 48). It is, therefore, suggested that researchers encourage individuals to participate in more MSE. For children and adolescents, engaging in more muscle promoting activity contributes to higher levels of muscular fitness, including muscular strength and endurance (13). This supports the current study findings that MSE is associated with muscular strength. Moreover, evidence has suggested that MSE can increase other physical fitness indicators. Morrow et al. (49) found that adherence to the MSE guideline was more likely to produce greater levels of cardiorespiratory fitness. The present study also provides evidence for supporting previous, well-recognised guidelines that children and adolescents should engage in MSE at least three times a week, as this study is the very first to examine the association between meeting the MSE guidelines and various physical fitness indicators (albeit self-reported). Collectively, promoting MSE in children and adolescents should be a priority for future health promotion initiatives.

In addition to cardiorespiratory fitness and muscle strength, the current study also suggests that participating in sports and muscle-strengthening exercises are positively associated with speed, agility, and flexibility. Currently, there is no evidence concerning the associations between these two types of physical activity with speed, agility, and flexibility, so it is impossible to find comparable evidence. One explanation for the research findings is that participating in sports and muscle-strengthening exercises might make children and adolescents feel fitter, leading them to report higher levels of these two attributes.

Somewhat inconsistently with previous studies (21), the results found that AC was not associated with any self-reported HRPF indicators. Indeed, as AC is associated with higher levels of physical activity, it is likely that AC may lead to greater physical fitness among children and adolescents. A systematic review found that AC was positively associated with cardiorespiratory fitness in children and adolescents (21). In contrast, the present study does not support this review. Also, the current study suggests that AC is not associated with other HRPF indicators. We assumed several possible reasons to interpret why AC is not associated with HRPF indicators, including the lower intensity of AC (e.g., walking) in children and adolescents; and self-reported HRPF is subject to measurement and recalls bias; and that the measurement of AC is not well-validated. For example, in specific, although children and adolescent actively commuted between school and home, its duration and intensity may be restricted, which may not trigger the threshold of increasing children and adolescents' fitness level. Owing to the rare comparable evidence in this study, more observational and intervention studies are needed to explore and further confirm the roles of AC in physical fitness in children and adolescents.

### Study limitations and strengths

This study has some limitations inherent in its design, measuring, and participants. First, owing to the study's cross-sectional design, the study could draw no causal conclusions. In other words, the directionality of the association between participating in sports and physical fitness indicators could not be determined. Second, this study employed self-reported measures to collect data on all the variables, which are subject to recall bias and social desirability of the participants. However, it should also be acknowledged that using objective measures would be offering a solution for studies with a large sample size. Third, as this study adopted a convenient and non-probabilistic sampling method, the research findings may be more regionally than nationally replicable. Finally, BMI was not included, which should be mentioned in the current study as a limitation. Given these limitations, future studies are encouraged to generate stronger evidence. Despite these limitations, this study still has some strengths. This study is the first to assess the associations between participating in sports and self-reported physical fitness indicators, which therefore broadens the literature. Also, the sample size in this study was large, so sufficient statistical power was achieved. Finally, this study controlled for many covariates to accurately estimate the association between participating in sports and self-rated physical fitness.

### Practical implications

- As self-reported physical fitness is recognised as an important marker of health status, a regular assessment or surveillance of self-reported physical fitness should be incorporated into a large health surveillance system.
- Encouraging participation in sports and MSE is recommended.

## Conclusion

This study offers some evidence concerning the associations between some types of physical activity and self-reported, health-related physical fitness in children and adolescents, highlighting the roles of participating in sports and muscle-strengthening exercises in improving self-reported physical fitness. Future studies should confirm or negate the present research findings.

## Data availability statement

The original contributions presented in the study are included in the article/supplementary materials, further inquiries can be directed to the corresponding author.

## Ethics statement

The study protocol and procedure were approved by the Institutional Review Board (IRB) of the Shanghai University of Sport with a Grant Number of 102772021RT071. Written informed consent to participate in this study was provided by the participants' legal guardian/next of kin.

## Author contributions

CS: writing—original draft. SC and JY: formal analysis. LW, KL, JH, HS, and SC: writing—review and editing. All authors contributed to the article and approved the submitted version.

## Funding

This study was supported by the National Social Science Fund of China (Grant No. 17BTY015).

## Conflict of interest

The authors declare that the research was conducted in the absence of any commercial or financial relationships that could be construed as a potential conflict of interest.

## Publisher's note

All claims expressed in this article are solely those of the authors and do not necessarily represent those of their affiliated organizations, or those of the publisher, the editors and the reviewers. Any product that may be evaluated in this article, or claim that may be made by its manufacturer, is not guaranteed or endorsed by the publisher.
